# Dynamic Response of Graphitic Flakes in Nematic Liquid Crystals: Confinement and Host Effect

**DOI:** 10.3390/nano7090250

**Published:** 2017-09-01

**Authors:** Weiwei Tie, Surjya Sarathi Bhattacharyya, Yuanhao Gao, Zhi Zheng, Eun Jeong Shin, Tae Hyung Kim, MinSu Kim, Joong Hee Lee, Seung Hee Lee

**Affiliations:** 1 Key Laboratory of Micro-Nano Materials for Energy Storage and Conversion of Henan Province, Institute of Surface Micro and Nano Materials, College of Advanced Materials and Energy, Xuchang University, Xuchang 461000, Henan, China; dwtie929@hotmail.com (W.T.); surjyasarathi@gmail.com (S.S.B.); gyh-2007@sohu.com (Y.G.); zhengzhi99999@gmail.com (Z.Z.); 2 Applied Materials Institute for BIN Convergence, Department of BIN Convergence Technology and Graduate School of Printable and Flexible Electronics, Chonbuk National University, Jeonju 561-756, Jeonbuk, Korea; zxcvb823@naver.com (E.J.S.); kth1811@naver.com (T.H.K.); mkim12@kent.edu (M.K.); jhl@jbnu.ac.kr (J.H.L.); 3 Asutosh College, 92, Shyamaprasad Mukherjee Road, Kolkata 700 026, West Bengal, India

**Keywords:** graphitic flakes, liquid crystal, Maxwell-Wagner polarization, dynamic response

## Abstract

Electric field-induced reorientation of suspended graphitic (GP) flakes and its relaxation back to the original state in a nematic liquid crystal (NLC) host are of interest not only in academia, but also in industrial applications, such as polarizer-free and optical film-free displays, and electro-optic light modulators. As the phenomenon has been demonstrated by thorough observation, the detailed study of the physical properties of the host NLC (the magnitude of dielectric anisotropy, elastic constants, and rotational viscosity), the size of the GP flakes, and cell thickness, are urgently required to be explored and investigated. Here, we demonstrate that the response time of GP flakes reorientation associated with an NLC host can be effectively enhanced by controlling the physical properties. In a vertical field-on state, higher dielectric anisotropy and higher elasticity of NLC give rise to quicker reorientation of the GP flakes (switching from planar to vertical alignment) due to the field-induced coupling effect of interfacial Maxwell-Wagner polarization and NLC reorientation. In a field off-state, lower rotational viscosity of NLC and lower cell thickness can help to reduce the decay time of GP flakes reoriented from vertical to planar alignment. This is mainly attributed to strong coupling between GP flakes and NLC originating from the strong *π*-*π* interaction between benzene rings in the honeycomb-like graphene structure and in NLC molecules. The high-uniformity of reoriented GP flakes exhibits a possibility of new light modulation with a relatively faster response time in the switching process and, thus, it can show potential application in field-induced memory and modulation devices.

## 1. Introduction

The importance of electric field-induced reorientation of non-spherical particles has gradually emerged, such that polymer cholesteric liquid crystal flakes (PCLC) for electro-optical devices and displays have been widely studied [1,2]. The PCLC flakes in a fluid opened a possibility to realize a particle-based and electric field-driven technology into a wide variety of applications such as displays and electronic papers with no optical polarizers and filters. Despite of the immense possibility of various applications, the development in switching technology of PCLC flakes is still in an early stage. For instance, the uniformity of reorientation needs to be improved and the relaxation time is required to be shortened.

Recently, an optically-opaque two-dimensional (2D) graphitic (GP) flake, which consists of multi-layered graphene sheets by *π*-*π* stacking, can provide an opportunity for a new light modulation technology [3,4,5]. Such technology can give rise to sufficient darkness in a field-off state due to the planar configuration of GP flakes in nematic liquid crystals (NLCs) with respect to the substrate. Under an applied electric field as low as tens of millivolts per micrometer, GP flakes reorient almost 90° from a planar face-up configuration to a perpendicular edge-up configuration, and a transmitting bright state is realized [5]. This switching technology upon electric field-induced reorientation of GP flakes relies on a combined interaction of the NLC director reorientation and Maxwell-Wagner polarization. Recent investigation over graphene oxide/thermotropic NLC-dispersed systems [6] has revealed electro-optical properties, viz. the threshold voltage and splay elastic constant of such systems strongly increase with increasing graphene oxide concentration. However, we have realized much reliable and reproducible switching technology for practical application devoid of electric field-induced flake aggregation. Information displays or electronic papers assisted by GP flake switching could realize low power consumption, high brightness, and multi-color capability without the use of filters and polarizers, which have great potential for both commercial and scientific applications.

The response time for reorientation of NLC can be described as:

(1) $$\tau_{decay}=\frac{\gamma_{1}d^{2}}{\pi^{2}K}=\frac{\gamma_{1}}{\varepsilon_{0}\left|\Delta \varepsilon \right|E_{c}^{2}}$$

and:

(2) τrise=γ1ε0|Δε|E2−(π2d2)K=γ1ε0|Δε|(E2−Ec2)

where *γ*_1_, *d*, *K*, *ε*_0_, and Δ*ε* denote physical properties of a liquid crystal, viz. the rotational viscosity, the cell thickness, the elastic constant, the dielectric constant in vacuum, and the dielectric anisotropy, respectively, and *E_c_*, and *E* denote the critical electric field and an external electric field, respectively [6,7,8,9,10]. This implies that reducing *γ*_1_ and *d*, and increasing *K* and Δ*ε* will contribute to a decrease in the response time of liquid crystals. GP flakes, while suspended in liquid crystal materials, reorient and relax following the liquid crystal director [5,10]. The simplified model to describe such an inhomogeneous configuration can be a double-layered structure with different dielectric permittivity *ε* and conductivity *σ* of each material, and the relaxation frequency can be described as [10]:

(3) $$\tau =\kappa \varepsilon_{0}\frac{\varepsilon_{1}+\varepsilon_{2}}{\sigma_{1}+\sigma_{2}}$$

This interaction can be further explained as the electric field-induced potential energy of GP flakes coupling with the contribution of positive NLC reorientation to generate the rotational kinetic energy for flake reorientation [5]. More importantly, the field-induced reorientation of GP flakes relaxes back to their initial planar face-up state for recovering the dark field-off state aided by the NLC director reorientation. This reversible reorientation of GP flakes allows optical modulation controlled by an electric field signal. Although the total response time, i.e., the time to complete both the reorientation and relaxation process is faster than PCLC flakes in general [1,2], the response time of the GP flakes still needs to be further improved for practical applications. We have carefully studied the dynamic reorientation phenomenon of graphene/graphitic flakes in NLCs under an electric field [5]. In the field-on state, we have found that the reorientation time of the GP device can be efficiently improved by optimizing several parameters, such as the electric field strength, the flake anisotropy, as well as the flake size and, again, the reorientation depends on the field-induced synergistic effect of interfacial Maxwell-Wagner polarization and NLC director reorientation. It is also found that *π*-*π* interaction between the GP surface and NLC molecule is so strong that the surface anchoring between the NLC molecules and GP flakes will allow such GP flakes following the NLC directors to reorient it collaboratively [7,8,11,12,13]. Thus, further investigation on the contribution of NLC director reorientation to the GP flake reorientation and the relaxation process is urgently required.

The response time of such system is defined in [5], and it can be further modified as:

(4) $$\tau \approx \frac{2\pi \sqrt{\rho a}R_{g}}{\sqrt{\left(\frac{4\pi }{3}l_{i}B_{i}E^{2}+2AK\right)-\frac{\gamma_{1}^{2}}{4\rho a{R_{g}}^{2}}}},$$

where notations represent that the mass per unit area of flakes *ρ*, the projected area of flakes *a*, radius of gyration *R_g_*, dimension of flakes in the axes (*i* = *x*, *y*, *z*) *l*, and a multiplicative constant *A*. The Clausius-Mosotti factor is defined by:

$$B_{i}=\frac{\left(\varepsilon_{p}-\varepsilon_{h}\right)}{\left[\varepsilon_{h}+L_{i}\left(\varepsilon_{p}-\varepsilon_{h}\right)\right]},$$

where *L_i_* is the geometrical depolarization factor. Therefore, variables in Equation (4), such as the physical properties of the NLC host, size, and aspect ratio of GP flakes, will provide a detailed contribution to the response time of the reorientation, as well as the relaxation process of the GP flakes.

## 2. Materials and Methods

The functionalized GP flakes have been synthesized through room-temperature oxidation of precursor graphite to generate graphite oxide and subsequent low-temperature thermal exfoliation to obtain functionalized graphene. The synthesized functionalized graphene mostly consists of an exfoliated graphene layer and some portion of the stacked layer structures with an expanded interlayer spacing of 7.5 Å. The named functionalized graphitic flake generated in the present synthesis scheme contains fewer defects over graphene sheets (relatively unbroken *sp*^2^ hybridization carbon atom over graphene plane) and less oxygen content over the graphene plane in comparison with commercial graphene oxide. The shape, uniformity, and internal structure of the flakes have been characterized using optical microscopy (OM) and scanning electron microscopy (SEM). The synthesis and detailed physical characterization of the GP flakes used in present investigation have been described elsewhere [3,14]. 

Test cells named as Case I, Case II and Case III shown in Table 1 were prepared in order to investigate the NLC host type and cell thickness-dependent reorientation and relaxation behavior of the GP flakes in NLC by comparing with the previous results as in [5]. Indium-tin-oxide (ITO) is used as electrodes on transparent glass substrates. Homogeneous alignment layers on both substrates are made by spin-coating a polyimide solution, viz. SE-6514 (Japan Synthetic Rubber Co., Tokyo, Japan), and then the substrates are baked at 70 °C for 5 min and 200 °C for one hour. The baked alignment layers are uniformly rubbed by a rubbing machine. The GP flakes and NLC suspension is sandwiched by the rubbed ITO substrates in anti-parallel direction and the uniform cell thickness is maintained at ~44 µm using a film spacer between the substrates. The cell thickness has been measured by comparing the capacitance of an empty cell and a benzene-filled cell using a 4284A Inductance capacitance resistance (LCR-meter) (Agilent Technologies, Santa Clara, CA, USA) [3,5,15].

Two commercially-available positive dielectric anisotropic NLC mixtures with different physical properties (dielectric anisotropy Δ*ε* = 5.3, rotational viscosity *γ* = 132 mPa·s, splay elastic constant *K*_11_ = 13.2 × 10^−12^ N), viz. ZLI-4792, and (Δ*ε* = 14.8, *γ* = 148 mPa·s, *K*_11_ = 9.3 × 10^−12^ N), viz. ZLI-4535 (Merck, Tokyo, Japan), have been used for present investigation. The GP flake-NLC suspensions have been prepared by adding ~0.005 wt % of flake powders directly into the NLC mixtures. In order to achieve the homogeneity of the dispersion of flakes with minimum damage, the suspensions are ultrasonicated for one hour, keeping the temperature at 30 °C (frequency, 28 kHz; input power, 60 W).

The used GP flake-NLC suspensions with ZLI-4792 and ZLI-4535 are filled into test cells and named as Case I, Case II, and Case III [5], respectively, as shown in Table 2. Samples are filled into the mentioned cells by capillary action at room temperature. Although most of the GP flakes in the GP-NLC suspension are supposed to be single-layered, or composed of a few layers of graphene after exfoliation, some thicker graphitic stacks do remain suspended in the mixture, exhibiting the desired dark state. They are characterized by optical microscopy, having significantly distinct thickness formed by several hundred layers forming an optically opaque state, and suspended in the planar face-up configuration in NLCs with an area of 10–300 μm^2^ and, hence, a volume of 10–450 μm^3^. The sample textures are captured by a Panasonic 5100 digital camera with a time resolution of 100 ms attached with optical microscope (OM, Nikon DXM1200, Tokyo, Japan) while test cells are driven by an AC field (0 to 200 mV/μm) with a constant driving frequency (60 Hz) using an arbitrary function generator (Tektronix AFG3022, Beaverton, OR, USA). The transmitted light intensity of the captured images has been analyzed with the help of IMT *i*-Solution software (Image and Microscope Technology Co.).

## 3. Results and Discussion

**Figure 1:**
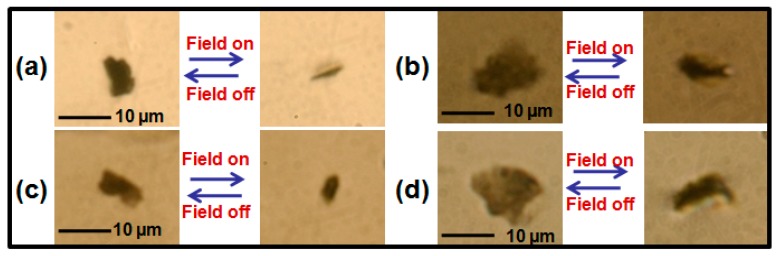
(Color online) Optical microphotographs exhibiting field-induced reorientation of representative GP flakes with similar flake areas and aspect ratios in low (**a**,**b**) and high (**c**,**d**) dielectric anisotropy nematic liquid crystal. (**a**–**d**) Case I: flake area and aspect ratio in (**a**,**b**): 35 μm^2^ with an aspect ratio of 2:1 and 85 μm^2^ with an aspect ratio of 1:1; Case II: flake area and aspect ratio in (**c**,**d**): 37 μm^2^ with an aspect ratio of 2:1 and 90 μm^2^ with an aspect ratio of 1:1.

The micrometer-sized GP flakes dispersed in the homogeneously-aligned NLC host in the mentioned cell geometry have been observed under an optical microscope. Figure 1 shows the representative case study of arbitrarily-shaped GP flakes’ reorientation for comparable morphology at field-off and field-on states with a fixed AC electric field strength of 90.0 mV/μm. Areas of suspended GP flakes in the field-off planar face-up configuration are microscopically measured to be 35 and 85 μm^2^ in the NLC host of ZIL4792 (a,b) (Case I) and those for the GP flake counterpart are 37 and 90 μm^2^ in the NLC host of ZIL4535 (c,d) (Case II), respectively. Figure 1 clearly displays all GP flakes reorient from face-up to almost edge-up configuration around their long axis parallel to the applied field direction at field-on state. Once the driving field is removed, the GP flakes relax back to their planar initial state. This observed reorientation behavior is reliable and reproducible under hundreds of trials. 

**Figure 2:**
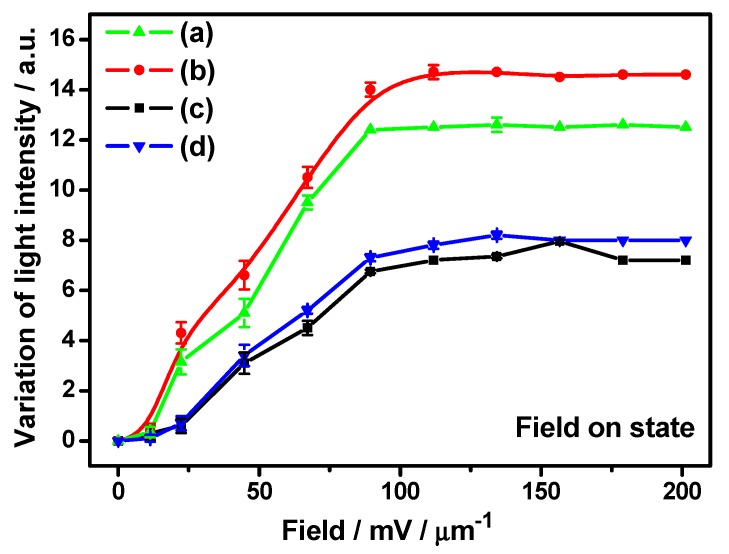
The measured reorientation-induced light intensity variation of representative GP flakes according to Figure 1. The applied electric fields for Case I and Case II are 0, 22, 44, 67, 90, 100, 112, 134, 156, 180, and 200 mV/μm, respectively (Case I: flake area and aspect ratio in (**a**,**b**): 35 μm^2^ with an aspect ratio of 2:1 and 85 μm^2^ with an aspect ratio of 1:1; Case II: flake area and aspect ratio in (**c**,**d**): 37 μm^2^ with an aspect ratio of 2:1 and 90 μm^2^ with an aspect ratio of 1:1). The scattered points represent experimental data along with measurement error bars and the lines are guides for the eye. Error bars for respective data points are assigned considering standard deviations obtained in the repetitive observations.

Figure 2 depicts the field-dependent variation of transmitted light intensity surrounding GP flakes. The optical transmittance has been measured locally on regions surrounding the GP flake in an area ~20 μm × 20 μm from optical microscopic images using *i*-Solution image analysis software and data points are plotted while the solid line represents a guide for the eye to the experimental data. Initially, transmission intensity is low as most of the transmission is blocked by the considerably large, effectively exposed area of the opaque flake in the direction of propagation of light. As the flakes reorient with the increasing applied field, the transmission gradually increases and, finally, reach their maximum transmittance for each case. Normally, a different maximum transmission intensity is ascribed to the different thicknesses of opaque graphitic flakes. In consideration of the field strength dependent reorientation time previously discussed in detail [5], we have investigated the influence of cell confinement on the response time of GP flakes in the next section. 

**Figure 3:**
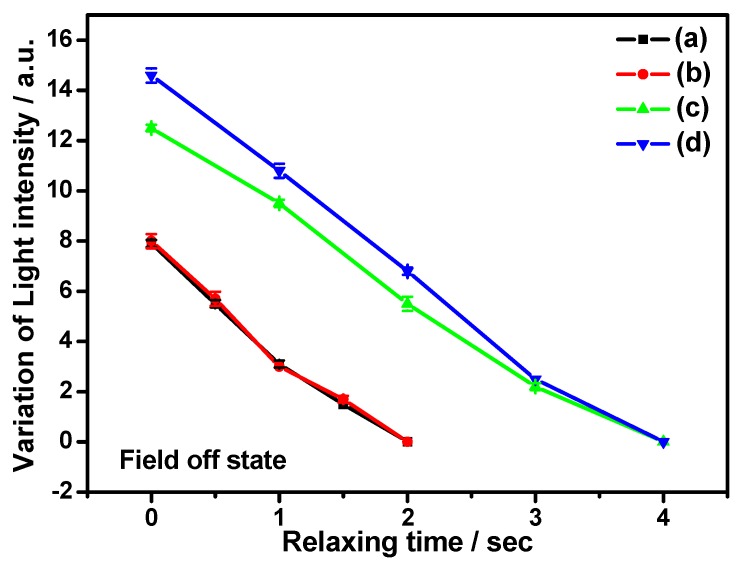
The measured relaxation-induced light intensity variation of representative GP flakes according to Figure 1. The field off state for Case I is 0 s, 0.5 s, 1 s, 1.5 s, and 2 s, and for Case II is 0 s, 1.0 s, 2.0 s, 3.0 s, and 4.0 s, respectively (Case I: flake area and aspect ratio in (**a**,**b**): 35 μm^2^ with an aspect ratio of 2:1 and 85 μm^2^ with an aspect ratio of 1:1; Case II: flake area and aspect ratio in (**c**,**d**): 37 μm^2^ with an aspect ratio of 2:1 and 90 μm^2^ with an aspect ratio of 1:1). The scattered points represent the experimental data along with measurement error bars, and the lines are guides for the eye. Error bars for respective data points are assigned considering standard deviations obtained in repetitive observations.

Figure 3 shows variation of transmitted light intensity as a function of relaxation time of functionalized graphene flakes in the LC matrix. It is interesting to mention here that the transmission intensity around GP flakes is found to be independent of the electric field. The flakes gradually recover to their initial state and, hence, the corresponding transmittance decreases after field removal. Experimental data is represented as scattered points with the solid line as a guide for the eye. However, it is observed that the flakes suspended in different NLC hosts recover to their initial state with different relaxation times, which will be mainly discussed in the following section. The mentioned observation confirms the reversible reorientation of GP flakes according to the optical images of Figure 1. Hence, the light transmission modulation is evidently controlled by GP flake reorientation and relaxation. Thus, electric field-controlled uniform reorientation and relaxation of GP flakes and the subsequent modulation of transmission intensity provide us a promising method for making new polarizer-independent electro-optic flake devices.

**Figure 4:**
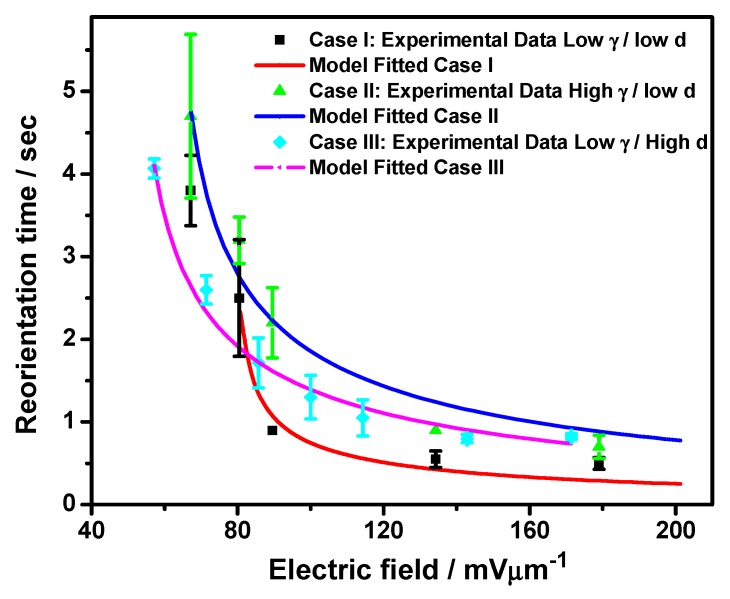
The averaged reorientation time of representative GP flakes with different NLC host-types and cell thicknesses as a function of the electric field (Case I: low *γ*, low *d*; Case II: high *γ*, low *d*; Case III: low *γ*, high *d*). The scattered points represents experimental data, along with measurement error bars and the lines, are model fitted with *Y* = *P*_1_/(*X*^2^ + *P*_2_)^0.5^ following Equation (4), where *P*_1_ is the numerator of Equation (4), and P_2_ is the constant part in the denominator of Equation (4) divided by coefficient of *E*^2^. Error bars for respective data points are assigned considering the standard deviations obtained of repetitive observations.

Figure 4 shows the NLC host-type and cell thickness-dependent reorientation time of GP flakes with comparable flake morphology under gradually-increasing AC electric field strength from 0 mV/µm to 200.0 mV/μm. It is worth mentioning here that the reported reorientation time has been measured from hundreds of case studies for flakes of several different shapes with aspect ratios from 1:1 to 1:3 and flake area in the range 20 μm^2^ to 90 μm^2^ while suspended in different NLC materials viz. ZIL4792 and ZIL4535, and variable cell confinement, i.e., cell thicknesses of 44 μm and 70 μm. Hence, we have classified numerous case studies for the reorientation time of GP flakes based on widely different dielectric anisotropy of the NLC host and cell confinement, i.e., the thickness of the used cells. Case I, Case II, and Case III of Figure 4 exhibit the reorientation time results in representative groups obtained under different experimental conditions with low dielectric anisotropy of the NLC host and low cell thickness, high dielectric anisotropy of the NLC host, and low cell thickness, low dielectric anisotropy of the host NLC, and high cell thickness, respectively. Reasonable differences obtained in the reorientation time of GP flakes in the NLC matrix for unique magnitudes of applied electric fields are represented by vertical error bars. Such variations are attributed to the differences in shape, area, and aspect ratio of the GP flakes suspended in the NLC matrix within the abovementioned range, as discussed in our earlier publication [5]. The electric field-dependent reorientation time of GP flakes shows a wide range of variation from 6.5 s to 1.5 s. Reorientation time of GP flakes in the NLC matrix clearly exhibits a decreasing trend with increasing applied electric field strength until saturation for all three cases. The vertical error bars for respective data points are assigned considering the standard deviations obtained through repetitive observations are also found to be significantly suppressed due to the high reproducibility of the recorded reorientation time at significantly higher fields. 

As the reorientation time of GP flakes in NLC with the field switched on is described in Equation (2), in Case I and Case II it is observed that strong dependence of the NLC host-types in identical cell confinement conditions exists. It is also found that GP flakes in NLC, viz. ZLI-4792 (Case I), having lower dielectric anisotropy (Δ*ε* = 5.3), lower rotational viscosity (*γ* = 132 mPa·s), and higher splay elastic constant (*K*_11_ = 13.2 × 10^−12^ N) reorient quicker than that in NLC, viz. ZLI-4535 (Case II), having higher dielectric anisotropy (Δ*ε* = 14.8), higher rotational viscosity (*γ* = 148 mPa·s), and lower (*K*_11_ = 9.3 × 10^−12^ N). The positive dielectric anisotropic NLC host experiences dielectric torque proportionate with the dielectric anisotropy of the NLC host under an oscillatory AC field. As the *π*-*π* interaction between the GP surface and NLC molecules is well known [5,6,7,8,11,12,13], NLC molecules adsorbed over the GP surface should induce a proportionate dielectric torque induced over the NLC host by the oscillating field to the suspended GP flakes. In addition, a comparable Maxwell-Wagner polarization is assumed to be induced over the GP surface under similar experimental conditions. Hence, GP flakes suspended in NLC, viz. ZLI-4535, should experience stronger dielectric torque in comparison with GP flakes suspended in NLC, viz. ZLI-4792, and should reorient faster. However, the larger rotational viscosity of ZLI-4535 hinders the reorientation process and GP flakes suspended in ZLI-4792 exhibit faster reorientation time, which agrees with our previous model that the rotational viscosity is one important factor for GP flakes’ reorientation time [5].

In order to determine the cell confinement conditions necessary for GP device application we have further examined the reorientation time of GP flakes in NLC, viz. ZLI-4792, with widely different cell thicknesses of ~44 µm (Case I) and 70 µm (Case III). The experimental results clearly suggest GP flakes suspended in thinner cells reorient faster than in thicker cells. The phenomenon might be anticipated from the considerable difference in the anchoring conditions of the NLC molecules in widely different cell thicknesses. The thicker cell (Case III) imposes weaker anchoring over NLC molecules, and dielectric torque-induced reorientation, and subsequent secondary-induced torque over suspended GP flakes remain weaker than its thinner counterpart (Case I and Case II). Hence, dependence of the reorientation time over the nature of NLC host properties is evident.

**Figure 5:**
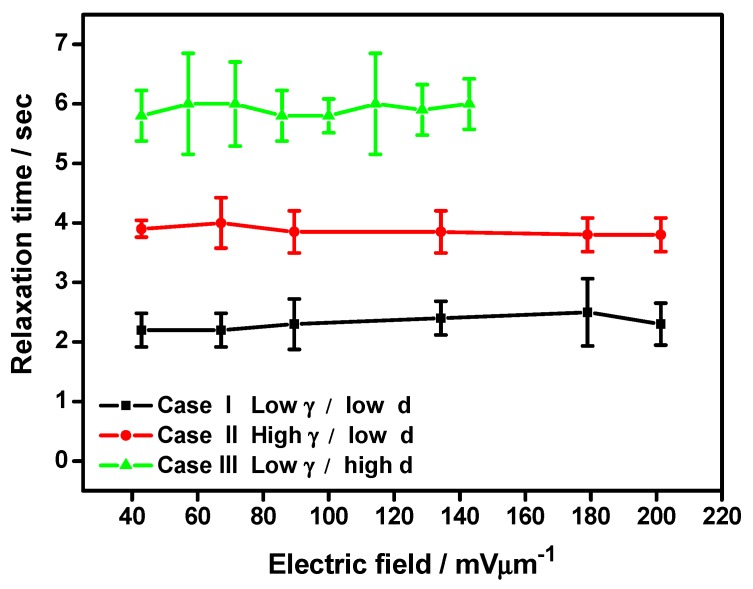
The relationship of the averaged relaxation time of representative GP flakes with different NLC host-types and cell thicknesses after removing the electric field (Case I: low *γ*, low *d*; Case II: high *γ*, low *d*; and Case III: low *γ*, high *d*). The scattered points represent the experimental data along with the measurement error bars, and the lines are guides for the eye. Error bars for respective data points are assigned considering the standard deviations obtained in repetitive observations.

For practical display applications the relaxation time of the GP device is also indispensable. The relaxation process of GP flakes in the NLC matrix from the field-aligned edge-up state to the planar face-up state after removing the electric field is recorded under an optical microscope. We have defined the relaxation time as the time taken by GP flakes to relax from the edge-up to the face-up configuration. Figure 5 shows the NLC host-type and cell thickness-dependent relaxation time of GP flakes with comparable flake morphology as a function of reorienting the AC electric field strength from 40 mV/μm to 200 mV/μm. Every case study of GP flakes’ field-off relaxation is followed by the abovementioned electric field-induced GP flake reorientation. Thus, an equivalent number of case studies in different NLC matrices and cell confinement with identical experimental conditions to the investigation of reorientation time is guaranteed. We have further regrouped the experimental relaxation time results similar to earlier demonstrated reorientation time cases. Hence, Case I, Case II, and Case III of Figure 5 exhibit the relaxation time results in representative groups obtained under different experimental conditions with low rotational viscosity of the NLC host and low cell thickness, high rotational viscosity of the NLC host, and low cell thickness, low rotational viscosity of the NLC host and high cell thickness, respectively. Reasonable differences are obtained in the relaxation time of GP flakes in the NLC matrix for distinctive magnitudes of the applied electric field, represented by vertical error bars derived from the standard deviations obtained in repetitive observations. Such variations are attributed to the differences in shape, area, and aspect ratio of GP flakes suspended in the NLC matrix within the previously-mentioned range, as discussed in our earlier publication [5]. The relaxation time of GP flakes is found to be independent of the applied reorienting field strength, as is the uncertainty in the relaxation time computation. However, relaxation time strongly depends on the confinement condition of the GP flakes in the NLC matrix, as well as the nature of the NLC host material. 

The GP flakes in NLC, viz. ZLI-4792 (Case I), having lower rotational viscosity (*γ* = 132 mPa·s) and higher splay elastic constant (*K*_11_ = 13.2 × 10^−12^ N) relax faster than that in NLC, viz. ZLI-4535 (Case II), having higher rotational viscosity (*γ* = 148 mPa·s) and lower (*K*_11_ = 9.3 × 10^−12^ N) in a similar confinement condition. The relaxation process of GP flakes in the NLC matrix is attributed to the relaxation of the NLC host. The *π*-*π* electron stacking between the graphene’s honeycomb structure and the NLC’s benzene rings results in a considerable amount of charge transfer and strong binding of NLC molecules over graphene surface with a binding energy of U_anchoring_ = −2 eV [7,8,13]. Density functional calculations further support the said facts [16]. In relaxation, this strong anchoring energy between NLC molecules and the graphene surface drives the GP flake to rotate following relaxation of the NLC director. However, the rotational viscosity of the NLC matrix hinders the relaxation process and, hence, faster relaxation time has been realized in the NLC host having lower rotational viscosity, viz. ZLI-4792 (Case I).

In addition, the cell thickness-dependent relaxation time of GP flakes with the same NLC host (Case I and Case III) has also been reported in Figure 5. Here, GP flakes’ relaxation time in NLC, viz. ZLI-4792, with 44 μm (Case I) has been compared to that in NLC, viz. ZLI-4792, with 70 μm (Case III), where the GP flakes with similar morphology have been tested [5]. The averaged relaxation time in Case I with lower d is found to be quicker than that in Case III. As mentioned earlier, the thinner cell imposes a stronger surface-induced anchoring over confined NLC molecules. Hence, NLC molecules adsorbed over the GP surface will induce stronger relaxing torque onto the GP flake due to the strong coupling of graphene/NLC than its thicker counterpart. Hence, dependence of the relaxation time over cell confinement conditions is evident. This observation is in compliance with the relaxation mechanism of NLC as described elsewhere [9,17,18,19]. 

## 4. Conclusions

We have demonstrated the electric field-induced dynamic reorientation and relaxation processes of GP flakes in an NLC host. The field-induced reorientation time of GP flakes has been effectively reduced by using the suitable physical properties of an NLC host. Our investigation reveals quicker reorientation time of GP flakes in the NLC host with higher *K*_11_ and lower *γ*_1_. Considering the flake reorientation phenomenon in positive dielectric anisotropy NLCs, the reorientation process depends not only on NLC director reorientation, but also the interfacial Maxwell-Wagner model. At the interface between GP flakes and NLC molecules, strong *π*-*π* interaction exists, and the relaxation process is owing to the elasticity-driven rotation of NLC director. The higher *K*_11_ and lower *γ*_1_ are responsible for higher elastic torque, which matches with the modified rising time equation well in conformity with our previous reported reorientation model. In a field-off state, the relaxation of GP flakes mainly follows the nematic NLC director that mechanically rotates after the field switches off. The GP flakes relax quicker in the NLC host with higher *K*_11_ and lower *γ*_1_ than its higher counterpart, and with lower *d* than its thicker counterpart, where the anchoring effect over the NLC director and subsequent secondary induced torque over suspended GP flakes are mainly responsible for this behavior. The reversible reorientation behavior of GP flakes in NLC and the improved response open up an efficient way of dynamically and quickly controlling transmittance properties of electro-optic devices, which makes them interesting candidates for potential applications.

## Figures and Tables

**Table 1:** Prepared test cells.

Samples	LC	Cell Thickness (μm)
Case I	ZLI-4792	44
Case II	ZLI-4535	44
Case III (from reference [5])	ZLI-4792	70

**Table 2:** Measured GP flakes of Case I, Case II and Case III.

Samples	Flake Area (μm^2^)	Flake Aspect Ratio (μm)
Case I	Case 1: 35	Case 1: 2:1
	Case 2: 85	Case 2: 1:1
Case II	Case 1: 37	Case 2: 2:1
	Case 2: 90	Case 2: 1:1
Case III (from reference [5])	Case 1: 65	Case 2: 2:1
	Case 2: 85	Case 2: 2.6:1
